# SNHG17 promotes colorectal tumorigenesis and metastasis via regulating Trim23-PES1 axis and miR-339-5p-FOSL2-SNHG17 positive feedback loop

**DOI:** 10.1186/s13046-021-02162-8

**Published:** 2021-11-15

**Authors:** Zehua Bian, Mingyue Zhou, Kaisa Cui, Fan Yang, Yulin Cao, Shengbai Sun, Bingxin Liu, Liang Gong, Jiuming Li, Xue Wang, Chaoqun Li, Surui Yao, Yuan Yin, Shenglin Huang, Bojian Fei, Zhaohui Huang

**Affiliations:** 1 Wuxi Cancer Institute, Affiliated Hospital of Jiangnan University, Wuxi, 214062 Jiangsu China; 2 Laboratory of Cancer Epigenetics, Wuxi School of Medicine, Jiangnan University, Wuxi, 214122 Jiangsu China; 3 Fudan University Shanghai Cancer Center and Institutes of Biomedical Sciences, Fudan University, Shanghai, 200032 China; 4 Department of Surgical Oncology, Affiliated Hospital of Jiangnan University, 200 Hui He Road, Wuxi, 214062 Jiangsu China

**Keywords:** CRC, lncRNA, SNHG17, PES1, FOSL2, miR-339-5p

## Abstract

**Background:**

Small nucleolar RNA host gene (SNHG) long noncoding RNAs (lncRNAs) are frequently dysregulated in human cancers and involved in tumorigenesis and progression. SNHG17 has been reported as a candidate oncogene in several cancer types, however, its regulatory role in colorectal cancer (CRC) is unclear.

**Methods:**

SNHG17 expression in multiple CRC cohorts was assessed by RT-qPCR or bioinformatic analyses. Cell viability was evaluated using Cell Counting Kit-8 (CCK-8) and colony formation assays. Cell mobility and invasiveness were assessed by Transwell assays. Tumor xenograft and metastasis models were applied to confirm the effects of SNHG17 on CRC tumorigenesis and metastasis in vivo. Immunohistochemistry staining was used to measure protein expression in cancer tissues. RNA pull-down, RNA immunoprecipitation, chromatin immunoprecipitation, and dual luciferase assays were used to investigate the molecular mechanism of SNHG17 in CRC.

**Results:**

Using multiple cohorts, we confirmed that SNHG17 is aberrantly upregulated in CRC and correlated with poor survival. In vitro and in vivo functional assays indicated that SNHG17 facilitates CRC proliferation and metastasis. SNHG17 impedes PES1 degradation by inhibiting Trim23-mediated ubiquitination of PES1. SNHG17 upregulates FOSL2 by sponging miR-339-5p, and FOSL2 transcription activates SNHG17 expression, uncovering a SNHG17-miR-339-5p-FOSL2-SNHG17 positive feedback loop.

**Conclusions:**

We identified SNHG17 as an oncogenic lncRNA in CRC and identified abnormal upregulation of SNHG17 as a prognostic risk factor for CRC. Our mechanistic investigations demonstrated, for the first time, that SNHG17 promotes tumor growth and metastasis through two different regulatory mechanisms, SNHG17-Trim23-PES1 axis and SNHG17-miR-339-5p-FOSL2-SNHG17 positive feedback loop, which may be exploited for CRC therapy.

**Supplementary Information:**

The online version contains supplementary material available at 10.1186/s13046-021-02162-8.

## Background

Colorectal cancer (CRC) is the third most prevalent malignant tumor and one of the leading causes of cancer-related death worldwide [1]. Despite progress in surgery, chemotherapy and radiotherapy, the prognosis of CRC is still poor due to its postoperative recurrence and metastasis. Therefore, it is urgent to explore the molecular mechanism of CRC and to develop new therapeutic targets.

Long noncoding RNAs (lncRNAs) are a class of transcribed RNAs with lengths greater than 200 nucleotides, and their protein coding ability is lost or restricted [2]. Recent studies have shown that lncRNAs play vital roles in regulating various biological processes, such as proliferation, differentiation, apoptosis, and chemoresistance [3]. Growing evidence has shown that the abnormal regulation or expression of lncRNAs is implicated in the tumorigenesis and progression of CRC [4, 5]. We have reported that some lncRNAs regulate CRC growth, metastasis and chemoresistance and may be potential prognostic biomarkers or therapeutic targets [6–12]. Recently, other groups also reported the regulatory roles of some lncRNAs, including ENO1-IT1 [13], LINC00460 [14], RAMS11 [15] and FLANC [16], in CRC development and progression. All these studies indicate the key regulatory roles of lncRNAs in CRC.

Small nucleolar RNAs (snoRNAs) are predominantly distributed in the nucleolus and play a role in guiding the sequence-specific chemical modification or processing of pre-ribosomal RNA [17]. As the host genes of snoRNAs, lncRNA small nucleolar RNA host genes (SNHGs) have been shown to be abnormally expressed in multiple cancers and regulate cell proliferation, metastasis, and chemoresistance [18, 19]. Of them, SNHG17 has been reported to be aberrantly overexpressed in multiple human cancers [20–25], suggesting that SNHG17 has extensive functions and universal roles in tumorigenesis. However, the biological function and mechanism of SNHG17 in CRC remain poorly understood. In this study, we demonstrated that SNHG17 is aberrantly overexpressed in CRC and correlated with poor clinical outcomes. We also uncovered, for the first time, that SNHG17 promotes tumor growth and metastasis via two different regulatory mechanisms, SNHG17-Trim23-PES1 axis and SNHG17-miR-339-5p-FOSL2-SNHG17 positive feedback loop, suggesting that SNHG17 could be a potential therapeutic target for CRC.

## Materials and methods

### Clinical samples

Human primary CRC tissues and their paired adjacent noncancerous tissues (NCTs) were obtained from the Affiliated Hospital of Jiangnan University and Fudan University Shanghai Cancer Center (Supplementary Table S1). All pathologically confirmed CRC tissue samples (cohort 1: NCTs = 51, CRC tissues = 91; cohort 2: NCTs = 107, CRC tissues = 107) were transferred to the laboratory in liquid nitrogen and stored at − 80 °C until use. Participants were excluded in the presence of any other malignancies or preoperative anti-cancer treatment. All patients signed informed consent forms, and the project was approved by the Clinical Research Ethics Committees of the participating institutions.

### Cell lines and culture

Human CRC cell lines HCT116, HCT8, SW620, HT29 and LoVo were purchased from the American Type Culture Collection (ATCC). LoVo cells were cultured in F12K medium, and the other cells were maintained in DMEM containing 10% fetal bovine serum. All these cell lines were cultured in 5% CO_2_ at 37 °C.

### Plasmids and transfection

siRNAs targeting SNHG17, PES1, and FOSL2 and miR-339-5p mimics and miR-339-5p inhibitor were ordered from Shanghai Genepharma Co., Ltd. Full-length SNHG17 was amplified and cloned into the pLenti-EF1a-EGFP-F2A-Puro-CMV-MCS vector using ClonExpress II One Step Cloning Kit (Vazyme, China). The short hairpin SNHG17-#2 was inserted into the pLKO.1 TRC cloning vector for lentiviral packaging. The promoter of SNHG17 was amplified from HCT116 genomic DNA by PCR and cloned into the pGL3-Basic vector. The related primers are listed in Supplementary Table S2. The transfection of these plasmids was conducted by using Lipofectamine 2000 (Invitrogen, USA) according to the manufacturer’s instructions.

### Quantitative reverse transcription PCR (RT-qPCR)

Total RNA of cells or tissue specimens was extracted using RNA isolate (Vazyme) and reverse transcribed into cDNA using a HiFiScript cDNA Synthesis Kit (CWBIO, China). Cytoplasmic and nuclear RNA isolations were performed with a PARIS Kit (Life Technologies, USA) following the manufacturer’s instructions. SNHG17 expression levels were measured by RT-qPCR using UltraSYBR Mixture (CWBIO) on a ViiA 7 Real-Time PCR System (Applied Biosystems, USA) with the program of 95 °C for 10 min, 40 cycles of 95 °C for 15 s and 60 °C for 1 min. The relative gene expression levels were calculated by the 2^-△△Ct^ method with ACTB as an internal control. The related primer sequences are listed in Table S2.

### Cell counting Kit-8 (CCK-8) and colony formation assays

Cell proliferation was measured by CCK-8 (Beyotime, China) and colony formation assays. For the CCK-8 assay, a total of 10 μL CCK-8 solution was added to each well of a 96-well plate at 1, 2, 3 and 4 days after transfection. The absorbance was measured at 450 nm with a microplate reader (BioTek Instruments). For the colony formation assay, approximately 800 HCT116 or 2000 LoVo cells were plated in 6-well plates and incubated at 37 °C for 2 weeks.

### Transwell assays

Approximately 1 × 10^5^ HCT116 cells or 1.5 × 10^5^ LoVo cells were added to the upper compartment of a Transwell chamber (Corning, USA). After 24 h of incubation, cells on the lower surface were fixed with 10% formaldehyde for half an hour and then stained with crystal violet for observation. In the invasion assay, Matrigel (BD, USA) was used to coat the Transwell chamber before the experiments.

### Xenograft mouse model

A total of 2.0 × 10^6^ SNHG17-overexpressing HCT116 cells or 3.5 × 10^6^ SNHG17-depleted LoVo cells and their respective control cells were subcutaneously injected into the different flanks of 4-week-old male BALB/c nude mice (Shanghai SLAC Laboratory Animal, China) (randomly divided into 2 groups, *n* = 5 for each group). The tumor size was measured every 3 days after the tumors became visible. For the in vivo metastasis model, 1.5 × 10^6^ SNHG17-overexpressing HCT116 cells or SNHG17-depleted LoVo cells were injected into 7-week-old male BALB/c nude mice (*n* = 5 for each group) via the tail vein. Five weeks after injection, the lung nodules of mice were observed to assess tumor metastasis. All animal experiments were approved by the Clinical Research Ethics Committees of Affiliated Hospital of Jiangnan University.

### Fluorescence in situ hybridization (FISH)

SNHG17 and 18S FISH probes were purchased from RiboBio. A FISH kit was employed to detect the signals of the probes according to the manufacturer’s protocol (RiboBio, China). The cells were fixed with 4% polyoxymethylene and incubated with permeabilizing solution (0.5% Triton X-100 diluted in PBS) at 4 °C for 5 min. After washing three times with PBS, the cells were treated with prehybridization buffer at 37 °C for 30 min. Then, a 20 μM probe mixture diluted in hybridization buffer was incubated with the cells overnight at 37 °C. Images of cells were captured after staining with DAPI dye for 10 min using an OLYMPUS DP80-Cellsens microscopic imaging system.

### Western blotting

The cells were lysed in RIPA buffer (Beyotime) supplemented with protease inhibitor cocktail (MCE, USA), and the obtained proteins were then separated by 10% SDS-PAGE and transferred to a PVDF membrane (Millipore, USA). After being blocked in 5% skimmed milk powder, the membranes were incubated with primary antibodies against PES1 (1:5000, Proteintech, USA), FOSL2 (1:1000, Cell Signaling Technology, USA), and GAPDH (1:5000, ABclonal, China) overnight at 4 °C. The protein band intensity was detected using a ChemiDOCTMXRS+ imaging system (BIO-RAD, USA).

### RNA pull-down assay

RNA pull-down assays were performed using the Pierce™ Magnetic RNA-Protein Pull-Down Kit (Thermo Fisher Scientific, USA) according to the manufacturer’s instructions as we previously described [8]. Detailed information regarding the primers used for in vitro transcription is depicted in Supplementary Table S2.

### RNA immunoprecipitation (RIP)

The EZ-Magna RIP kit (Millipore) was used for RIP assays according to the manufacturer’s instructions. HCT116 cell lysates were incubated overnight at 4 °C in RIP buffer containing magnetic beads conjugated to anti-PES1 or anti-IgG as a control. The lysates were then treated with proteinase K buffer, followed by RNA extraction. Finally, the purified RNA was examined by RT-qPCR to detect the abundance of SNHG17.

### Dual luciferase reporter assay

Luciferase reporter vectors containing SNHG17 and FOSL2 with wild-type (WT) or mutated (MUT) miR-339-5p binding sites were constructed. Luciferase reporter plasmids were cotransfected with miR-339-5p mimics or miR-NC mimics into 293 T and HCT116 cells by Lipofectamine 2000. The luciferase activities of these cells were detected at 48 h after transfection using a Dual-Luciferase Reporter Assay System (Beyotime).

### Chromatin immunoprecipitation (ChIP)

CRC cells were preserved with formaldehyde and fixated for 10 min to produce DNA-protein cross-links. Then, ChIP assays were performed using ChIP assay kits (Beyotime) according to the manufacturer’s instructions. The cell lysates were sonicated to produce chromatin fragments of 200–400 bp, which were immunoprecipitated with FOSL2 (Cell Signaling Technology) or IgG (Beyotime) antibodies. Precipitated chromatin DNA was recovered and analyzed by PCR. The primers used for the promoters are listed in Supplementary Table S2.

### Immunohistochemistry (IHC)

The slides of the tissue microarray were incubated with the primary antibody for PES1 (1:100, Proteintech) or FOSL2 (1:100, Cell Signaling Technology) overnight at 4 °C. IHC was performed as we described previously [8].

### Statistical analyses

The data were analyzed by GraphPad Prism version 8.0 (GraphPad Prism) and SPSS 20 software (SPSS). All results are presented as the mean ± SD. Student’s t test and χ2 test were used to assess the significance of differences between groups. The differences in survival rates were determined with the Kaplan-Meier method and compared with the log-rank test. *P* < 0.05 was considered to indicate statistical significance.

## Results

### SNHG17 is frequently upregulated in CRC and inversely associated with patient survival

**Fig. 1:**
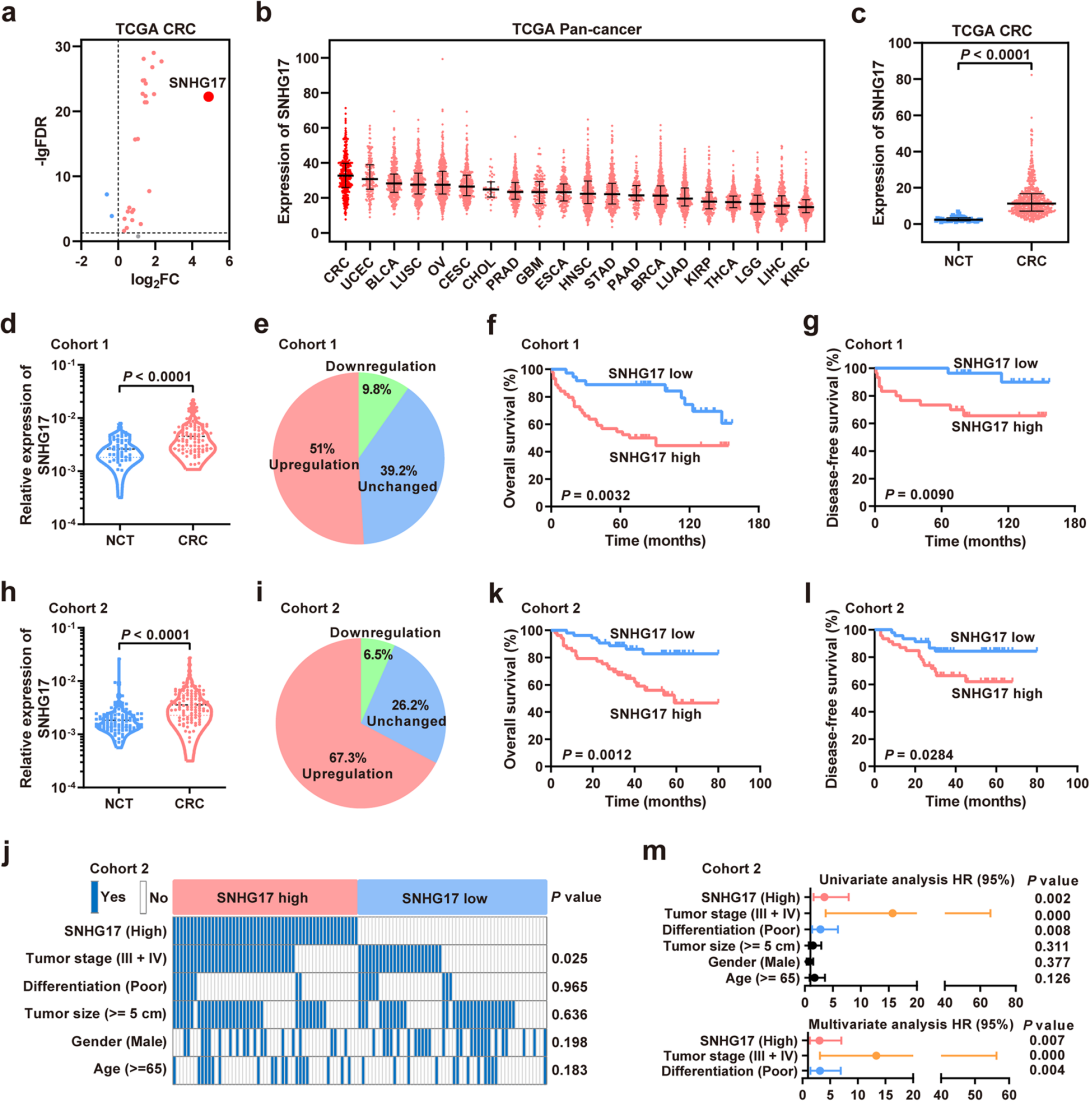
SNHG17 is upregulated in CRC tissues, and its high expression predicted a poor prognosis. **a** The expression of all SNHGs in CRC tissues of the TCGA database. **b** The expression of SNHG17 in the pan-cancer TCGA dataset. **c** The expression of SNHG17 in CRC tissues and NCTs of the TCGA database. **d** RT-qPCR analysis of SNHG17 expression in CRC tissues from cohort 1. **e** The percent change in SNHG17 expression in cohort 1 CRC tissues compared with NCTs. **f, g** Kaplan-Meier survival analyses of the overall survival (**f**) and disease-free survival (**g**) in CRC cohort 1 with different expression levels of SNHG17. **h** The relative expression of SNHG17 in CRC cohort 2. **i** The rate of SNHG17 upregulation, downregulation and unchanged expression in 107 paired CRC tissues and NCTs. **j** The relationship between the SNHG17 expression level and clinical pathological parameters of CRC. **k, l** Kaplan-Meier survival analyses of overall survival (**k**) and disease-free survival (**l**) in CRC cohort 2 patients with low or high expression of SNHG17. **m** Univariate and multivariate regression analyses of independent prognostic factors in CRC patients.

SNHGs have been shown to be abnormally expressed in multiple cancers. To determine the expression of the SNHG family in CRC, we checked the expression of all SNHGs in CRC tissues of The Cancer Genome Atlas (TCGA) database and confirmed that most SNHGs were upregulated in CRC (Fig. 1a). Of these SNHGs, SNHG17 showed the highest expression level in CRC (Fig. 1a). SNHG17 was also confirmed to be upregulated across cancers in the TCGA database, with the highest expression in CRC (Fig. 1b and c). In addition, SNHG17 was upregulated in several CRC Gene Expression Omnibus (GEO) datasets (Supplementary Fig. 1). The expression of SNHG17 was further examined in cell lines of different cancers from the Cancer Cell Line Encyclopedia (CCLE) database. As expected, SNHG17 was highly expressed in CRC cell lines (Supplementary Fig. 2). All these results suggest that SNHG17 is a key oncogenic lncRNA in CRC.

First, we validated the overexpression of SNHG17 in CRC cohort 1 (NCTs = 51, CRC tissues = 91) by RT-qPCR and revealed that SNHG17 was upregulated in 51% (26/51) of CRC tissues compared with their paired NCTs (Fig. 1d and e). We then used the Kaplan-Meier method and log-rank test to determine the potential effect of SNHG17 expression on patient prognosis. The results showed that patients with high SNHG17 expression levels exhibited poor overall survival (log rank = 8.669, *P* = 0.0032, Fig. 1f) and poor disease-free survival (log rank = 6.821, *P* = 0.0090, Fig. 1 g). Survival analyses also revealed that patients with relatively high expression of SNHG17 had shorter OS times than patients with relatively low expression level in CRC cohorts from TCGA and GSE41258 (Supplementary Fig. 3).

In addition, these findings were further validated in an independent CRC cohort (NCTs = 107, CRC tissues = 107) and showed that SNHG17 was upregulated in 67.3% (72/107) of CRC tissues compared with their paired NCTs (Fig. 1 h and i) and was positively correlated with tumor stage (Fig. 1j). Survival analyses confirmed that patients with high SNHG17 expression levels exhibited poorer overall survival (log rank = 10.57, *P* = 0.0012, Fig. 1 k) and disease-free survival (log rank = 4.803, *P* = 0.0284, Fig. 1 l) than those with low expression levels. Furthermore, univariate and multivariate Cox proportional hazard analyses identified that SNHG17, tumor stage and differentiation degree were independent prognostic factors for CRC patients (Fig. 1 m).

### SNHG17 promotes CRC growth and metastasis

**Fig. 2:**
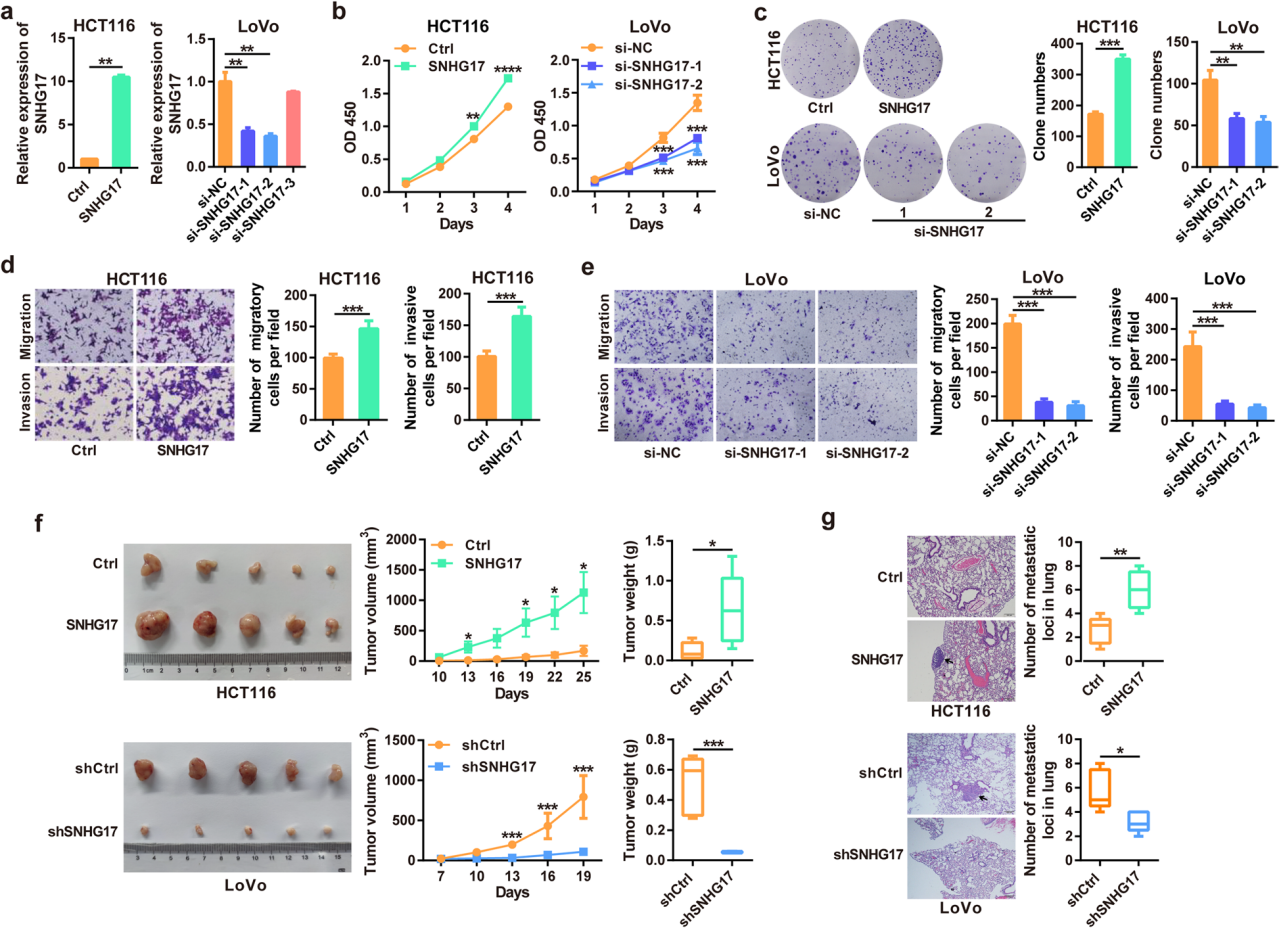
SNHG17 promotes CRC proliferation and metastasis in vitro and in vivo. **a** The overexpression and knockdown efficiency of SNHG17 was detected by RT-qPCR after transfection with SNHG17 expression plasmid and three different SNHG17 siRNAs. **b, c** CCK-8 and colony formation assays were applied to detect the cell proliferation ability of HCT116 and LoVo cells after SNHG17 overexpression or knockdown. **d, e** Transwell assays were used to assess the migration and invasion abilities of HCT116 and LoVo cells after SNHG17 overexpression (**d**) or knockdown (**e**). **f** HCT116 cells stably overexpressing SNHG17 and SNHG17-depleted LoVo cells were subcutaneously injected into nude mice (*n* = 5) to evaluate the tumor formation ability in vivo. The tumor volume was measured every three days after tumors appeared, and the tumor weight was recorded after the mice were sacrificed. **g** HCT116 cells with SNHG17 overexpression and LoVo cells with SNHG17 knockdown were injected into the tail veins of nude mice (*n* = 5) to assess the metastasis ability of SNHG17. The number of lung nodules was counted after HE staining.

To determine the functional role of SNHG17 in CRC, we first assessed the expression levels of SNHG17 in different CRC cell lines (Supplementary Fig. 4). Then, gain- and loss-of-function studies of SNHG17 were carried out in HCT116 and LoVo cells, respectively (Fig. 2a). We found that overexpression of SNHG17 significantly promoted, whereas silencing of SNHG17 reduced, the cell proliferation and colony forming abilities of CRC cells (Fig. 2b and c). We further explored the effects of SNHG17 on CRC metastasis and showed that ectopic SNHG17 expression significantly promoted the migration and invasion of HCT116 cells, whereas SNHG17 knockdown inhibited the migration and invasion of LoVo cells (Fig. 2d and e). Consistent with these results, in vivo studies showed that ectopic expression of SNHG17 promoted CRC tumor growth, whereas SNHG17 knockdown inhibited CRC tumor growth in nude mice (Fig. 2f). In addition, we assessed the impact of SNHG17 on metastasis in vivo using a lung metastasis mouse model. The results revealed that SNHG17 overexpression significantly promoted CRC pulmonary metastasis, whereas SNHG17 knockdown inhibited CRC pulmonary metastasis (Fig. 2 g). Collectively, these data demonstrate that SNHG17 promotes CRC growth and metastasis.

### SNHG17 interacts with PES1 in CRC cells

**Fig. 3:**
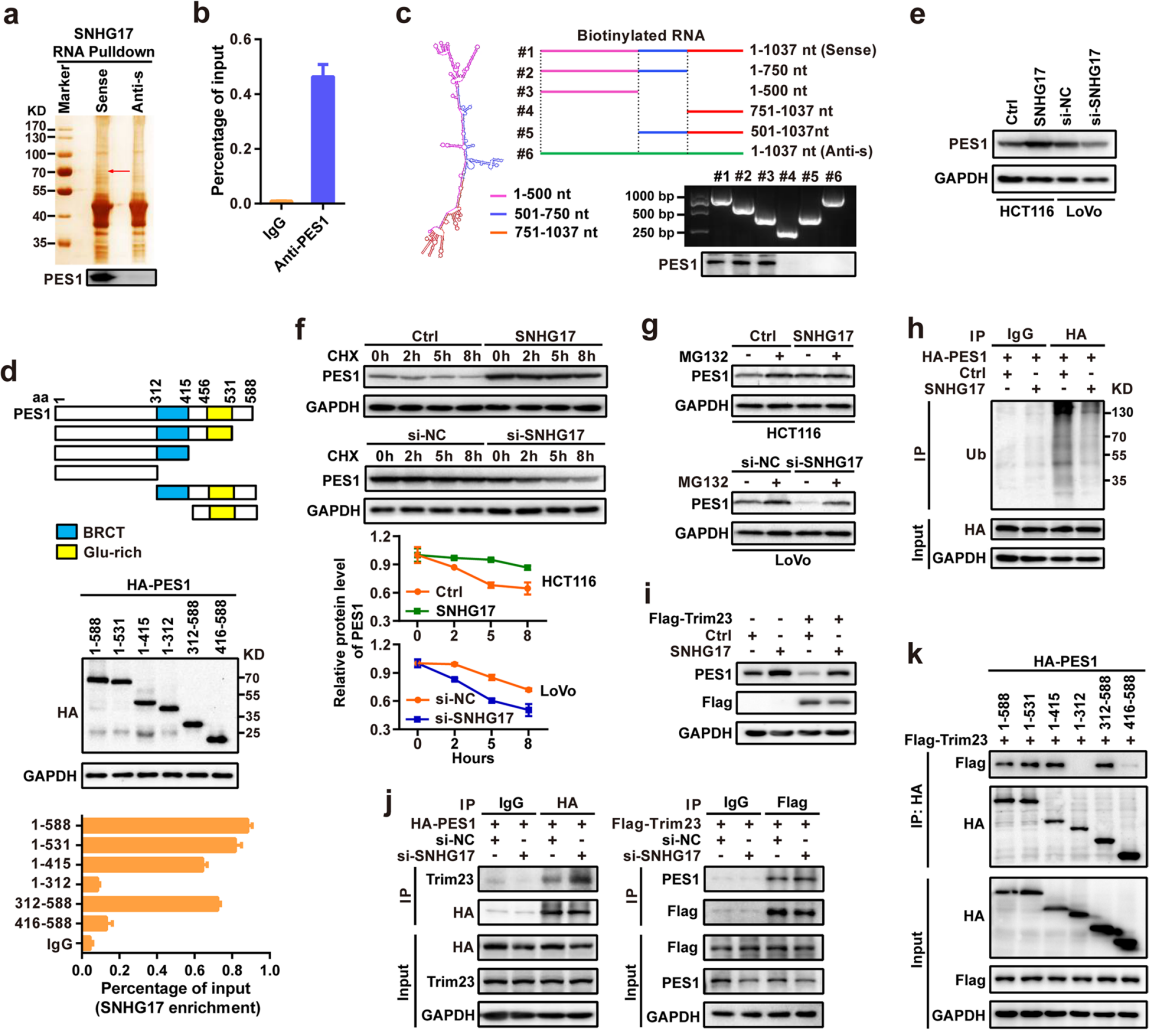
SNHG17 interacts with PES1 and blocks ubiquitin/proteasome-dependent PES1 degradation in CRC cells. **a** RNA pull-down assays followed mass spectrometry analysis indicated that SNHG17 could bind to PES1. **b** RIP assays using an antibody against PES1 followed by RT-qPCR to determine the enrichment of SNHG17. **c** A series of truncated SNHG17 fragments were used for RNA pulldown assays to detect the binding domain of SNHG17 on PES1. **d** Deletion mapping for the domains of PES1 that bound to SNHG17. **e** Western blotting for the protein levels of PES1 after SNHG17 overexpression or knockdown. **f** SNHG17 regulated PES1 stability. HCT116 cells and LoVo cells were treated with CHX at 50 μg/ml and harvested for western blotting assays at the indicated time points. **g** Cells with SNHG17-overexpression or knockdown were treated with MG132 (20 μM) for 6 h, and then western blotting was performed to detect PES1 levels. **h** Ub-Flag and PES1-HA were transfected into HCT116 cells with SNHG17 overexpression. The cells were treated with MG132 for 6 h prior to lysis. Immunoprecipitation was performed with an anti-HA antibody, and western blotting analyses were performed with an anti-Ub antibody. **i** The effects of SNHG17 and/or Trim23 overexpression on the protein levels of PES1 in HCT116 cells. **j** SNHG17 knockdown increased the association between PES1 and Trim23. The PES1-HA plasmid or Trim23-Flag plasmid was co-transfected into si-NC- or si-SNHG17-transfected LoVo cells. **k** A series of truncated PES1 mutants were used for IP assays to detect the binding domain of PES1 on Trim23. The cells were treated with MG132 for 6 h prior to lysis.

To further identify protein targets directly regulated by SNHG17, we performed RNA pull-down assays to identify SNHG17-associated proteins in CRC cells. The retrieved proteins were subjected to SDS-PAGE, and several additional differential bands were selected for mass spectrum analyses (Fig. 3a). Based on the functional annotation of proteins predicted by mass spectrum analyses, PES1 was selected as a potential SNHG17-associated protein. Western blotting assays further confirmed the binding of SNHG17 to PES1 using the retrieved proteins in the RNA pull-down assay (Fig. 3a). Moreover, RNA immunoprecipitation (RIP) assays performed with an anti-PES1 antibody versus an IgG antibody showed significant enrichment of SNHG17 (Fig. 3b). Taken together, these data suggest that SNHG17 physically associates with PES1.

To identify which region of SNHG17 binds to PES1, we constructed a series of SNHG17 deletion mutants based on the predicted secondary structure of SNHG17 in the AnnoLnc2 database (http://annolnc.gao-lab.org/index.php). RNA fragments were transcribed in vitro from these deletion mutant constructs and used for RNA pull-down assays. Western blotting analyses of PES1 in protein samples pulled down by these different SNHG17 constructs showed that RNA fragments with the 1–500 deletion nearly completely lost their ability to bind PES1, suggesting that the N-terminus of SNHG17 is essential for the binding of SNHG17 to PES1 (Fig. 3c). To further investigate which domain of PES1 accounts for its interaction with SNHG17, we performed RIP assays using a series of HA-tagged PES1 deletion mutants. As shown in Fig. 3d, the BRCT domain of PES1 is essential for the binding of PES1 to SNHG17 (Fig. 3d).

### SNHG17 blocks PES1 ubiquitination and degradation

Given that SNHG17 interacts with the PES1 in CRC cells, we next investigated the molecular consequence of this association on PES1 expression. The results showed that the protein levels of PES1 were significantly increased in SNHG17-overexpressing HCT116 cells and decreased in SNHG17-silenced LoVo cells (Fig. 3e), whereas no obvious effect was observed on the mRNA levels of PES1 (Supplementary Fig. 5). These results suggest that SNHG17 can increase PES1 protein expression at the posttranscriptional level. To further validate this observation, we used the protein synthesis inhibitor cycloheximide (CHX) to evaluate the effect of SNHG17 on the protein stability of PES1. SNHG17 markedly increased the half-life of PES1 in HCT116 cells, whereas silencing SNHG17 expression reduced the half-life of PES1 degradation in LoVo cells (Fig. 3f). Moreover, treatment with the proteasome inhibitor MG132 attenuated the accumulation of endogenous PES1 in SNHG17-overexpressing cells and the reduction in PES1 expression in SNHG17 knockdown CRC cells (Fig. 3 g). These data suggest that SNHG17 interferes with PES1 degradation through the ubiquitin-proteasome system. Furthermore, the ubiquitination levels of PES1 significantly decreased in SNHG17-overexpressing cells (Fig. 3 h).

A recent study revealed that Trim23 is an E3 ligase for PES1 ubiquitination and degradation [26]. To investigate whether SNHG17 inhibits the effect of Trim23 on the degradation of PES1, we co-transfected SNHG17 and Trim23 expression vectors into CRC cells, and showed that Trim23 overexpression could decrease PES protein level, while SNHG17 overexpression could restore the decreased PES1 protein level caused by Trim23 overexpression (Fig. 3i). Next, we examined the effect of SNHG17 on the interaction between Trim23 and PES1 in CRC cells using IP assays. Indeed, SNHG17 knockdown notably increased this association in CRC cells, suggesting that SNHG17 increases the protein stability of PES1 by inhibiting Trim23-mediated ubiquitination of PES1 (Fig. 3j). We have confirmed that the BRCT domain of PES1 is crucial for the binding of PES1 to SNHG17 (Fig. 3d), which suggests that SNHG17 can compete with Trim23 to bind the BRCT domain of PES1, thereby inhibits Trim23-mediated ubiquitination and degradation of PES1. Therefore, we performed IP assays using a series of HA-tagged PES1 deletion mutants. As shown in Fig. 3 k, the BRCT domain of PES1 is also essential for the binding of PES1 to Trim23. Together, these results demonstrate that SNHG17 increases the protein levels of PES1 by binding PES1 and then inhibiting Trim23-mediated ubiquitination and degradation of PES1.

### SNHG17 binds with miR-339-5p

**Fig. 4:**
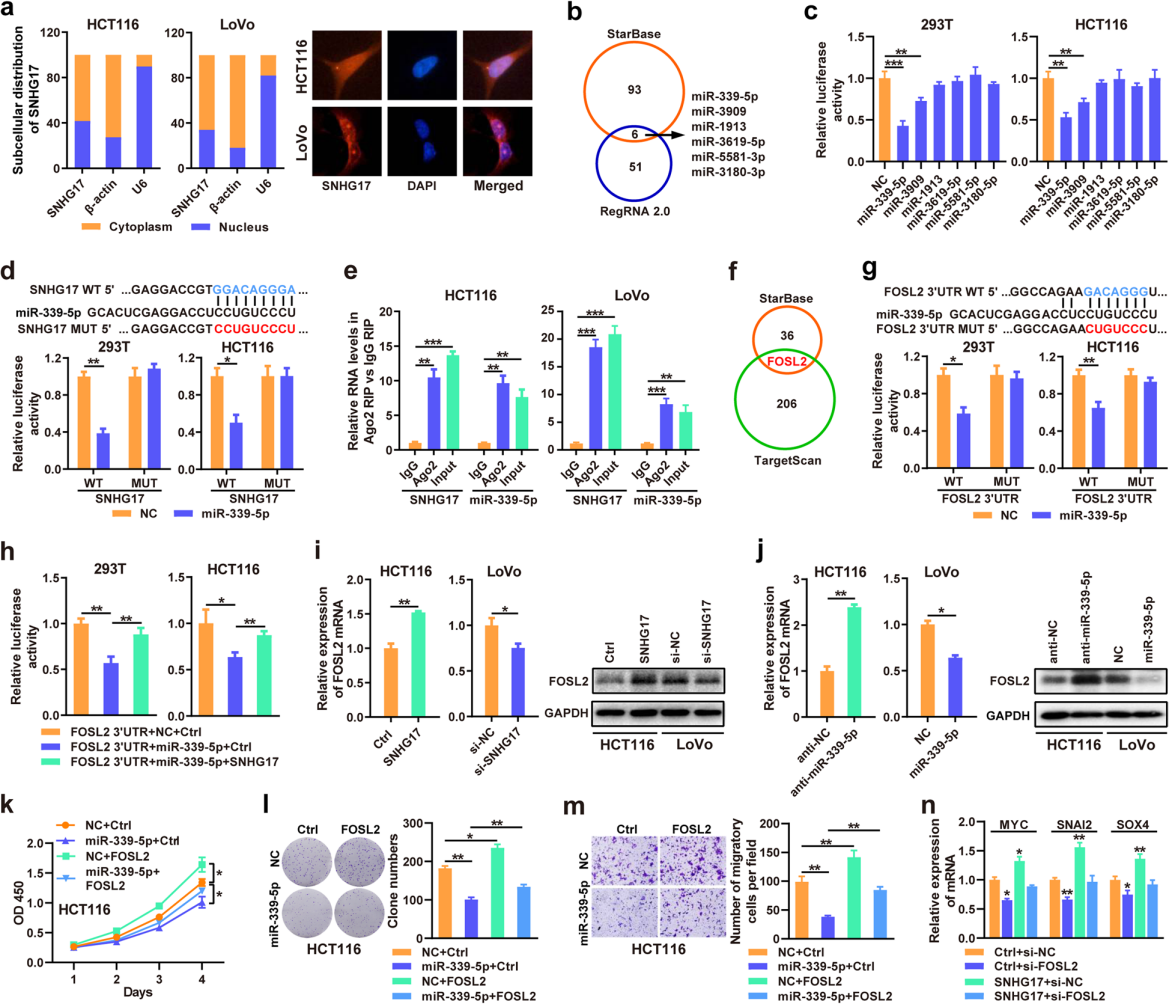
SNHG17 upregulates FOSL2 by binding with miR-339-5p**. a** The subcellular localization of SNHG17 was detected by RT-qPCR and FISH in HCT116 and LoVo cell lines. **b** StarBase and RegRNA 2.0 were used to predict SNHG17-associated miRNAs. **c** The luciferase activity of SNHG17 after cotransfection with miRNAs was determined by dual luciferase assays. **d** The relative luciferase activity of cells cotransfected with SNHG17-WT or SNHG17-MUT and miR-339-5p mimic was determined in 293 T and HCT116 cells by luciferase reporter assays. **e** Cellular lysates from HCT116 and LoVo cells were used for RIP with Ago2 antibody and IgG antibody. The levels of SNHG17 and miR-339-5p were detected by RT-qPCR. **f** The StarBase and TargetScan databases were used to predict the targets of the SNHG17/miR-339-5p axis. **g** Luciferase reporter assays were used to determine the relative luciferase activity of FOSL2–3′ UTR-WT or FOSL2–3′ UTR-MUT after cotransfection with miR-339-5p mimics. **h** MiR-339-5p and pluc-FOSL2–3′ UTR-WT were cotransfected with SNHG17 to verify whether SNHG17 can function as a ceRNA of miR-339-5p. **i** The mRNA and protein levels of FOSL2 were determined in SNHG17-overexpressing or SNHG17-depleted CRC cells by RT-qPCR and western blotting. **j** The mRNA and protein levels of FOSL2 were determined in miR-339-5p-depleted or miR-339-5p-overexpressing CRC cells by RT-qPCR and western blotting. **k-m** CCK-8 (**k**), colony formation (**l**) and Transwell (**m**) assays were applied to detect the cell proliferation and migration abilities of HCT116 cells transfected with miR-339-5p and FOSL2 expression vector. **n** The mRNA expression levels of FOSL2 target genes were determined in HCT116 cells transfected with FOSL2 siRNA and/or SNHG17 expression plasmid by RT-qPCR.

Numerous studies have demonstrated that cytoplasmic lncRNAs post-transcriptionally regulate downstream genes by binding with microRNAs (miRNAs). We detected the subcellular localization of SNHG17 using RT-qPCR and FISH assays and demonstrated that SNHG17 was located in both the cytoplasm and nucleus of HCT116 and LoVo cells (Fig. 4a and Supplementary Fig. 6). We predicted miRNAs that could regulate SNHG17 using StarBase and RegRNA 2.0 tools and identified six candidate miRNAs (miR-339-5p, − 1913, − 3619-5p, − 3180-3p, − 3909, and − 5581-3p) (Fig. 4b). Subsequently, we confirmed that miR-339-5p and miR-3909 could bind to SNHG17 through luciferase assays, and miR-339-5p had the strongest inhibitory effect on SNHG17 (Fig. 4c). We also showed that miR-339-5p was significantly downregulated in CRC tissues from the TCGA CRC and GEO GSE30454 datasets (Supplementary Fig. 7). Therefore, miR-339-5p was chosen for further studies. We constructed luciferase reporter vectors of SNHG17 segments containing WT or MUT miR-339-5p binding sites. Luciferase reporter assays revealed that miR-339-5p significantly decreased the reporter activity of the SNHG17-WT plasmid, whereas no obvious changes were observed in the mutant group, indicating that SNHG17 could specifically interact with miR-339-5p (Fig. 4d). Furthermore, the RIP assay showed that both SNHG17 and miR-339-5p were enriched in Ago2-containing miRNA ribonucleoprotein complexes (miRNPs) (Fig. 4e).

### FOSL2 is a functional target of SNHG17/miR-339-5p axis

Next, to identify the downstream target of the SNHG17/miR-339-5p axis, we analyzed the mRNA targets of the SNHG17-ceRNA network predicted by StarBase and the target genes of miR-339-5p predicted by TargetScan and identified that FOSL2 is a potential downstream target of the SNHG17/miR-339-5p axis in CRC (Fig. 4f). To further verify our speculation, we performed luciferase reporter assays in 293 T and HCT116 cells and showed that miR-339-5p significantly inhibited the reporter activity of the FOSL2 WT 3′ untranslated region (UTR) group but not that of the MUT group (Fig. 4 g). To further evaluate the relationships among SNHG17, miR-339-5p and FOSL2, pluc-FOSL2–3′ UTR-WT plasmids were cotransfected with miR-339-5p and SNHG17 into CRC cells for a luciferase assay, and the results indicated that ectopic SNHG17 expression could block the downregulation of luciferase activity induced by miR-339-5p (Fig. 4 h). Moreover, both the RNA and protein expression levels of FOSL2 were significantly increased in SNHG17-overexpressing HCT116 cells and decreased in SNHG17-depleted LoVo cells compared with their corresponding control cells (Fig. 4i). In addition, we revealed that the miR-339-5p inhibitor could increase the expression of FOSL2 compared with that in the control group, whereas ectopic miR-339-5p expression in CRC cells significantly decreased the expression of FOSL2 (Fig. 4j). Additional rescue assays confirmed that ectopic FOSL2 expression partly restored the cell proliferation and migration activities, which were decreased by miR-339-5p in CRC cells (Fig. 4 k-m). Moreover, SNHG17 overexpression partly restored the expression of the FOSL2 target genes (MYC, SNAI2 and SOX4) impaired by FOSL2 knockdown in HCT116 cells (Fig. 4n). All these results suggest that FOSL2 is a direct functional target of the SNHG17/miR-339-5p axis in CRC cells.

### SNHG17 overexpression is driven by FOSL2

**Fig. 5:**
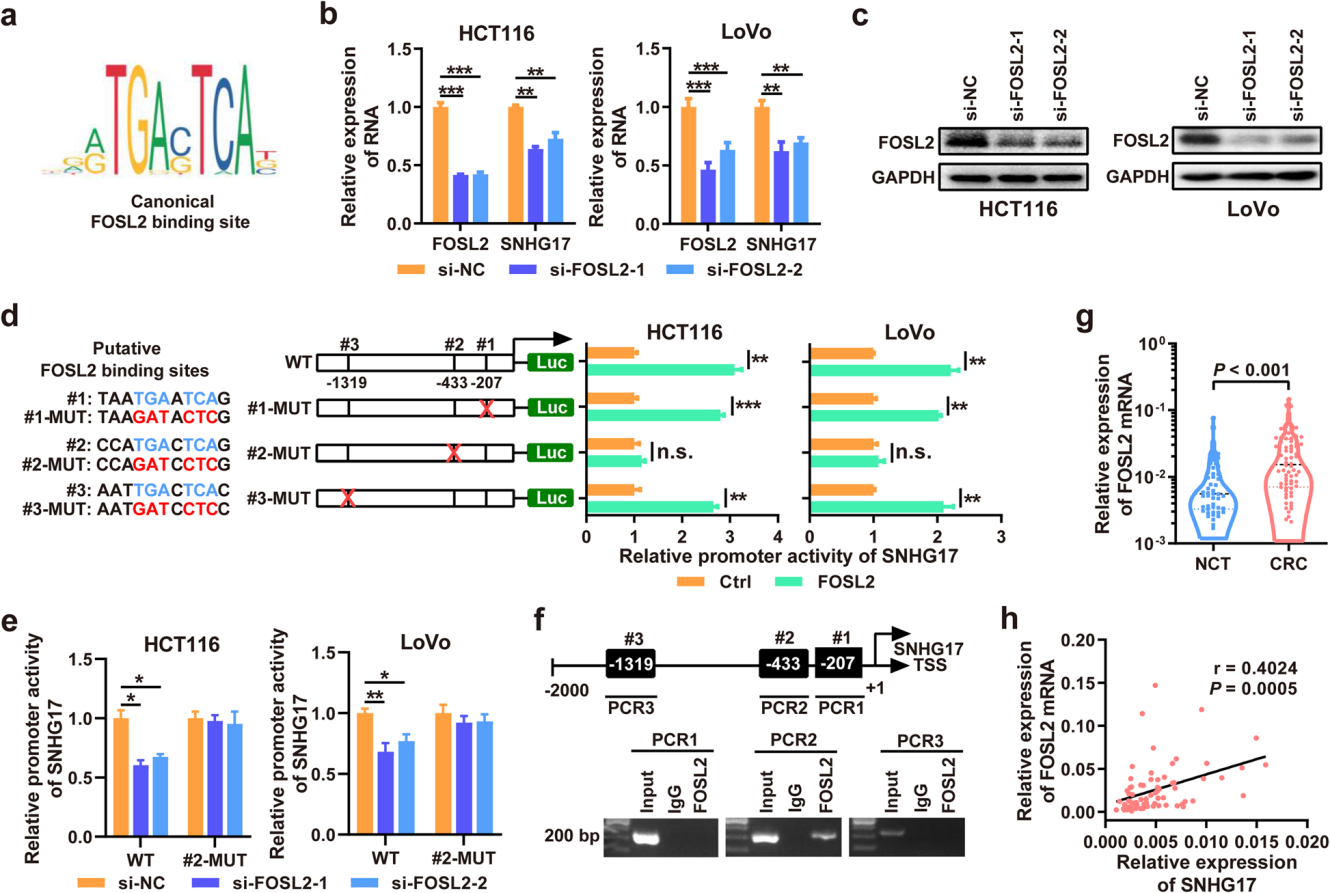
FOSL2 binds to the promoter of SNHG17 and promotes its expression. **a** The JASPAR database was used to search the FOSL2 binding motif within the SNHG17 promoter. **b** RT-qPCR was used to verify the expression of SNHG17 and FOSL2 when FOSL2 was silenced. **c** The knockdown efficiency of FOSL2 was detected by western blotting. **d** The luciferase activity of the SNHG17 promoter (WT, #1-MUT, #2-MUT and #3-MUT) was tested via luciferase reporter assays when FOSL2 was overexpressed. Schematic depictions show the different luciferase reporter plasmids; blue represents the predicted binding sites between FOSL2 and the SNHG17 promoter; and red represents the mutant sites. **e** The luciferase activity of the SNHG17 promoter (WT and #2-MUT) was tested via luciferase reporter assays when FOSL2 was silenced. **f** ChIP assays were conducted to prove that FOSL2 could bind to the SNHG17 promoter in HCT116 cells. **g** The relative mRNA expression of FOSL2 was verified in cohort 1 CRC tissues and NCTs by RT-qPCR. **h** Bivariate correlation analysis between SNHG17 RNA expression and FOSL2 mRNA expression in cohort 1 CRC tissues.

The mechanism mediating SNHG17 overexpression in CRC remains unclear. We scanned the binding sites of transcription factors on the regulatory element of SNHG17 by employing the JASPAR database. Interestingly, we observed several FOSL2 binding sites at the promoter of SNHG17, suggesting direct regulation of SNHG17 by FOSL2 (Fig. 5a). To validate our hypothesis, we knocked down the expression of FOSL2 in CRC cells with siRNAs and found that inhibiting FOSL2 expression significantly decreased the expression of SNHG17 (Fig. 5b and c). We then performed a luciferase assay to investigate whether FOSL2 directly regulates the transcription of SNHG17. We searched for the canonical binding site of FOSL2 predicted by JASPAR in the promoter region of SNHG17, and the three sites with the highest scores were selected for experimental verification. After mutating all three potential binding sites, luciferase assays suggested that the second binding site is the key site mediating the transcriptional regulation of SNHG17 by FOSL2 (Fig. 5d). To further substantiate this, we carried out additional luciferase reporter assays in FOSL2-depleted CRC cells. The results showed that FOSL2 knockdown reduced the activity of the WT SNHG17 promoter, which was abolished when site 2 was mutated (Fig. 5e). Moreover, ChIP assays confirmed the interaction between FOSL2 and putative binding site 2 in the SNHG17 promoter (Fig. 5f). We also examined the mRNA levels of FOSL2 in CRC tissues and revealed the upregulation of FOSL2 in CRC (Fig. 5 g). Moreover, Pearson correlation analysis showed that the expression of FOSL2 was positively correlated with that of SNHG17 in CRC tissues (Fig. 5 h, *r* = 0.4024, *P* = 0.0005). Together, these data demonstrate that FOSL2 directly drives SNHG17 overexpression in CRC by transcriptional activation.

### PES1 and FOSL2 are upregulated in CRC and positively correlated with SNHG17 expression

**Fig. 6:**
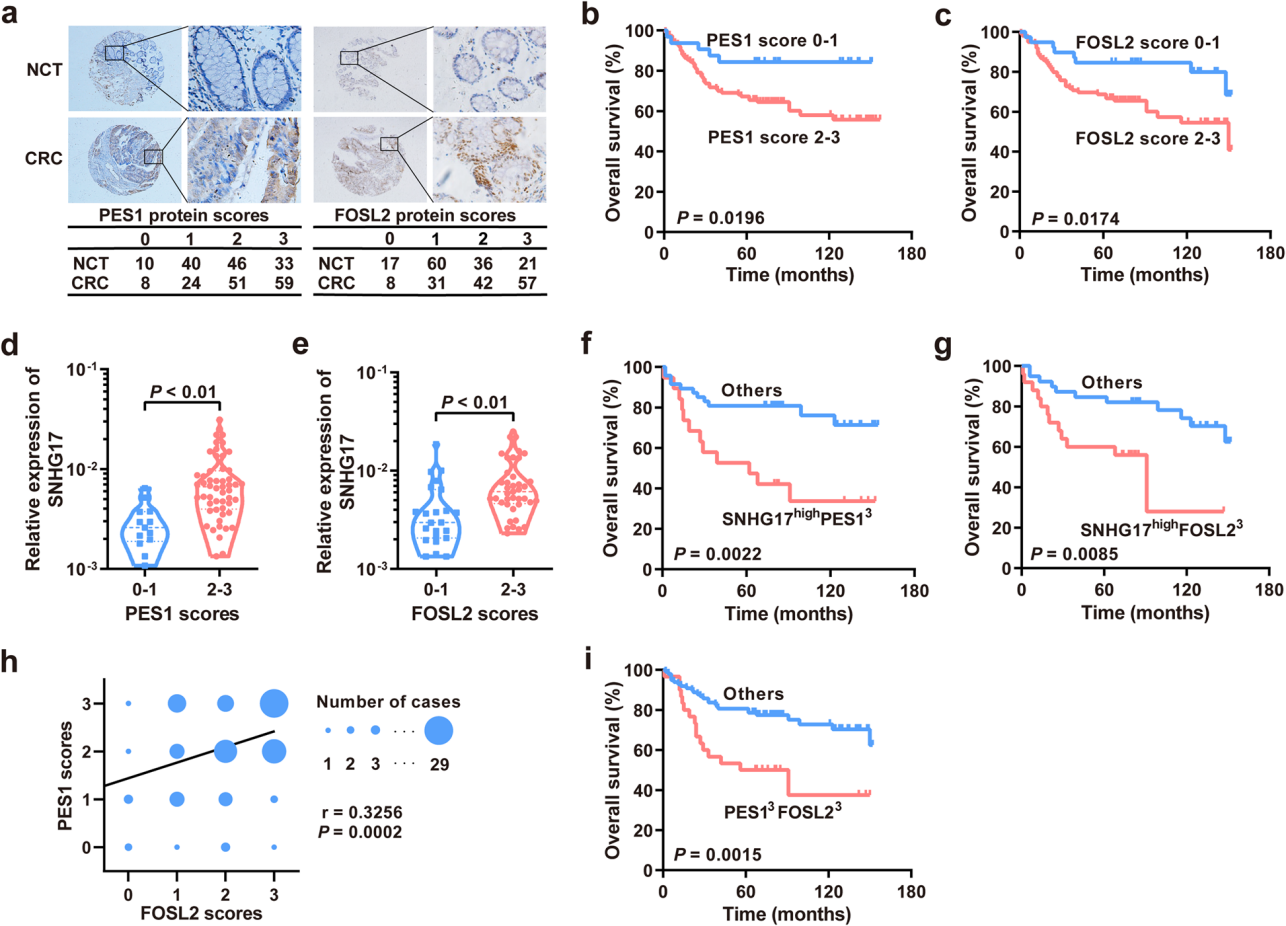
The protein expression of PES1 and FOSL2 is upregulated in CRC tissues. **a** Immunohistochemistry staining of PES1 and FOSL2 in CRC tissues and NCTs. **b, c** Kaplan-Meier survival analysis of CRC patients based on the protein expression scores of PES1 (**b**) or FOSL2 (**c**). **d, e** The relative expression of SNHG17 in two groups of CRC tissues with high and low protein levels of PES1 (**d**) or FOSL2 (**e**). **f, g** Combined influence of SNHG17 and PES1 (**f**) or FOSL2 (**g**) on the risk of CRC-related death using Kaplan-Meier survival analysis. **h** Bivariate correlation analysis between FOSL2 and PES1 protein expression in CRC tissues. **i** Combined influence of FOSL2 and PES1 on the risk of CRC-related death according to Kaplan-Meier survival analysis.

To further confirm the relationships among SNHG17 and PES1 and FOSL2, the protein expression of PES1 and FOSL2 in CRC tissues was assessed using IHC. The results indicated that tumor tissues showed increased PES1 and FOSL2 expression compared with NCTs (Fig. 6a). Survival analyses revealed that high protein expression of PES1 or FOSL2 was closely related to poor survival (log rank = 5.446, *P* = 0.0196, Fig. 6b) (log rank = 5.657, *P* = 0.0174, Fig. 6c). Moreover, the protein expression of both PES1 and FOSL2 in human CRC tissues was positively correlated with the expression of SNHG17 (Fig. 6d and e). Meanwhile, the positive relationship between SNHG17 and PES1 (or FOSL2) levels was also observed in CRC xenografts in nude mice (Supplementary Fig. 8). The prognosis of CRC patients with high expression of SNHG17 and PES1 (FOSL2) was significantly worse than that of other CRC patients (log rank = 9.410, *P* = 0.0022, Fig. 6f) (log rank = 6.933, *P* = 0.0085, Fig. 6 g). We further showed that the protein levels of PES1 were positively correlated with those of FOSL2 in CRC tissues (*r* = 0.3256, *P* = 0.0002, Fig. 6 h), and high expression of both PES1 and FOSL2 in CRC tissues indicated a poor prognosis in CRC patients (log rank = 10.09, *P* = 0.0015, Fig. 6i). In summary, these data suggest that PES1 and FOSL2 are candidate downstream targets of SNHG17.

### SNHG17 plays a carcinogenic role in CRC through PES1 and FOSL2

**Fig. 7:**
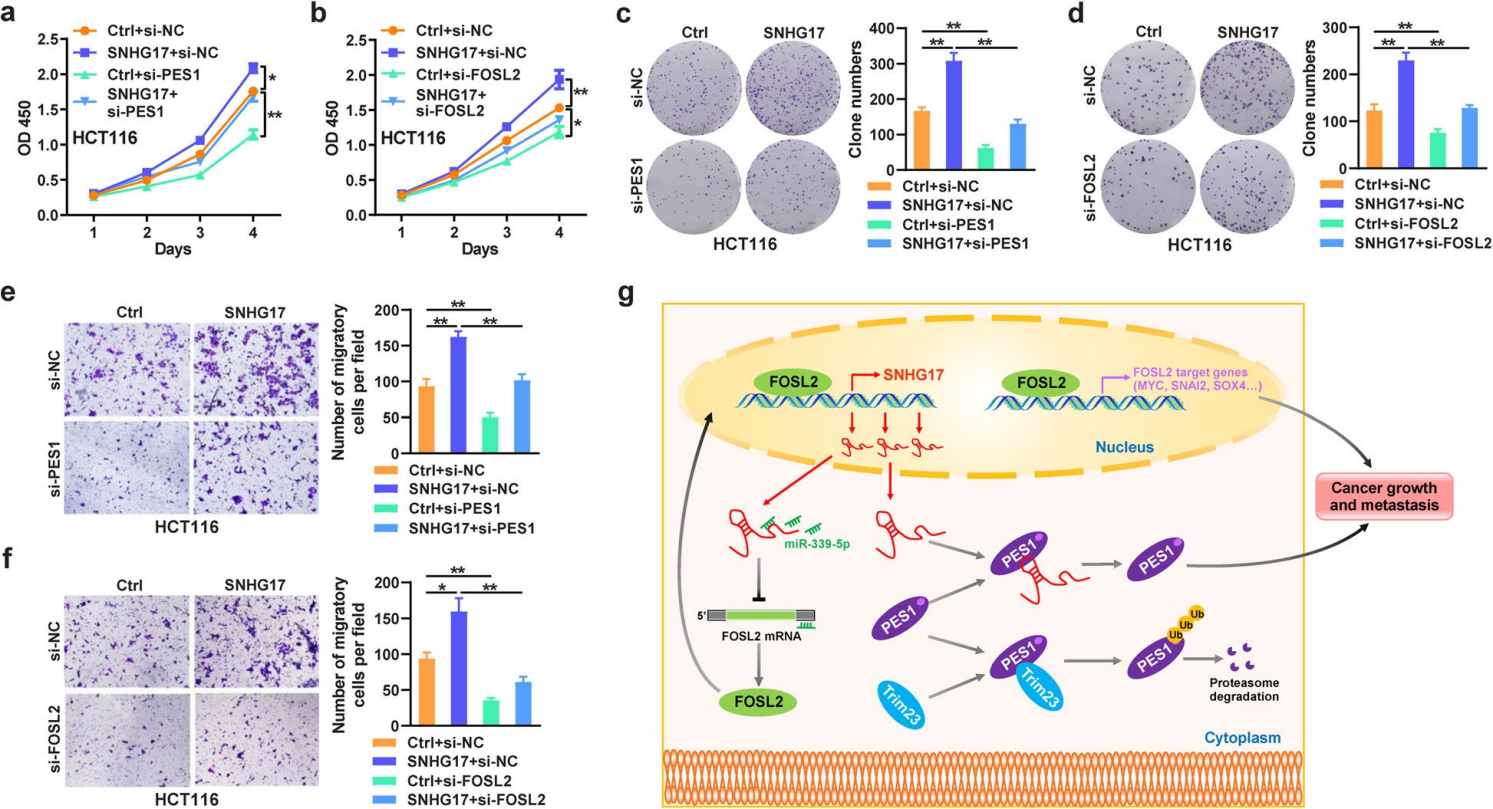
SNHG17 exerts its carcinogenic role in CRC through PES1 and FOSL2. **a, b** CCK-8 assays were used to detect the rescuing effects of SNHG17 overexpression on cell proliferation ability in PES1 (**a**)- or FOSL2 (**b**)-silenced HCT116 cells. **c, d** Colony formation assays were used to detect the rescuing effects of SNHG17 overexpression on colony formation ability in PES1 (**c**)- or FOSL2 (**d**)-silenced HCT116 cells. **e, f** Transwell assays were performed to evaluate the rescuing effects of SNHG17 overexpression on cell migration in PES1 (**e**)- or FOSL2 (**f**)-silenced HCT116 cells. **g** Integrated model depicting the oncogenic effects and mechanism of SNHG17 in terms of CRC growth and metastasis.

To investigate whether SNHG17 regulates CRC development and progression through PES1 and FOSL2, we designed rescue assays. It was confirmed that both PES1 and FOSL2 could inhibit cell proliferation in CRC cells, whereas the impact of SNHG17 on CRC cell proliferation was abolished by PES1 or FOSL2 knockdown (Fig. 7a-d). PES1 or FOSL2 knockdown also partly blocked the effects of SNHG17 overexpression on the migration of CRC cells (Fig. 7e and f). Collectively, we found that SNHG17 exerts tumor-promoting functions through PES1 and FOSL2 (Fig. 7 g).

## Discussion

CRC is one of the most common malignant tumors of the digestive system. LncRNAs can act as oncogenes or tumor suppressors, and their dysregulation is highly associated with the development and progression of CRC. SNHG17 was initially confirmed to be highly expressed in CRC, and then its overexpression was observed in a variety of cancers. Recent studies have indicated the oncogenic role of SNHG17 in several cancer types, including CRC. For example, recent studies showed that SNHG17 promotes tumor cell proliferation and invasion in tongue squamous cell carcinoma and CRC by sponging miR-23a-3p and miR-876, respectively [25, 27]. In addition, SNHG17 aggravates prostate cancer progression by positively regulating its homolog SNORA71B [28]. All these data suggest that SNHG17 is a pan-cancer oncogene and may be a promising therapeutic target across cancers.

In this study, we confirmed that SNHG17 is significantly upregulated in CRC tissues and was associated with poor survival in multiple CRC cohorts. Functionally, SNHG17 can promote CRC proliferation and metastasis. SNHG17 exerts tumor-promoting functions by two different mechanisms. We demonstrated that PES1 is a SNHG17-associated protein. PES1 is highly evolutionarily conserved and is involved in ribosome biogenesis and cell proliferation [29]. The ribosome is an important organ that is responsible for protein synthesis. To meet the needs for the continuous growth of tumor cells, it is necessary to increase ribosome biogenesis to maintain high protein synthesis efficiency. Therefore, increased ribosome biogenesis is an important feature of cancer cells [30]. Wang et al. reported that PES1 promotes tumorigenesis in hepatocellular carcinoma by regulating the PI3K/AKT pathway [31]. It has been reported that PES1 interacts with BRD4 to enhance c-Myc expression, thereby promoting cell growth and cell resistance to extra-terminal inhibitors in pancreatic cancer [32]. In addition, a recent study revealed that PES1 facilitates telomerase assembly and negatively correlates with senescence in cancer cells, suggesting that PES1 is a promising target for cancer therapy [33]. All these data suggest that PES1 plays an oncogenic role in various cancers. In this study, we showed that SNHG17 binds to PES1 and increases its protein expression in CRC cells. Mechanistically, SNHG17 interacts with PES1 to inhibit the Trim23-mediated ubiquitination of PES1, resulting in increased PES1 stability and enhanced tumor growth and metastasis. Clinical analyses revealed that PES1 is significantly upregulated in CRC tissues and predicts a poor prognosis. These findings suggest that targeting the SNHG17-PES1 regulatory axis is a promising strategy for CRC treatment.

SNHG17 is mainly located in cytoplasm. Most cytoplasmic lncRNAs act as miRNA sponges and are involved in miRNA-mediated posttranscriptional regulation. SNHG17 has already been confirmed to facilitate cell growth by modulating the miR-384/ELF1 axis in oral squamous cell carcinoma [34]. SNHG17 can promote tumor-like behavior in hepatocellular carcinoma cells via miR-3180-3p/RFX1 [35]. These results suggest that binding with miRNAs is one of the most important mechanisms of SNHG17 in cancer. In addition to the SNHG17-Trim23-PES1 mechanism, we also verified that SNHG17 could bind with miR-339-5p and inhibit its function. Previous studies have demonstrated that exosome-derived miR-339-5p mediates radiosensitivity by inhibiting Cdc25A [36]. MiR-339-5p has also been reported to inhibit glycolysis and colon cancer growth by reducing PKM2 expression through hnRNPA1 and PTBP1 [37]. In addition, we confirmed the tumor-suppressive effect of miR-339-5p in CRC and found that it was inhibited by SNHG17.

We further searched for the targets of SNHG17/miR-339-5p and revealed that FOSL2 is a novel functional target of miR-339-5p. Subsequent functional experiments confirmed that SNHG17 regulates CRC development and progression by competitively sponging miR-339-5p and restoring the activity of FOSL2. FOSL2 and the oncoproteins Fos and Jun belongs to the activator protein 1 transcription factor family that transactivates the transcription of its downstream targets. Aberrantly increased expression of FOSL2 has been documented in many cancer types, including CRC. Previous studies have shown that FOSL2 promotes the proliferation, migration, and invasion of various cancers, including non-small-cell lung cancer [38], hepatocellular carcinoma [39], and CRC [40]. We also observed that FOSL2 is upregulated and positively correlates with SNHG17 expression in CRC tissues, which is positively associated with and predicts poor clinical outcomes. Interestingly, we identified a putative binding site of FOSL2 at the promoter of SNHG17 and uncovered that FOSL2 transcriptionally activates SNHG17 expression by binding to this site. Collectively, our data revealed a positive feedback loop of SNHG17-miR-339-5p-FOSL2-SNHG17 in CRC and suggest that targeting this loop might be a promising strategy for CRC therapy.

## Conclusion

Overall, our study uncovered two different molecular mechanisms by which SNHG17 promotes CRC development and progression. SNHG17, miR-339-5p, PES1, FOSL2 and their downstream targets form a complicated regulatory network that contributes to colorectal tumorigenesis and metastasis. These data may inspire the development of conceptually novel cancer therapeutics.

## Supplementary Information

**Additional file 1.**

## Data Availability

The datasets used and/or analyzed during the current study are available from the corresponding author on reasonable request.

## Abbreviations

CRC: Colorectal cancer
LncRNAs: Long noncoding RNAs;
SNHG: Small nucleolar RNA host gene
CCK-8: Cell Counting Kit-8
ATCC: American Type Culture Collection
RT-qPCR: Quantitative reverse transcription PCR
FISH: Fluorescence in situ hybridization
RIP: RNA immunoprecipitation
ChIP: Chromatin immunoprecipitation
IHC: Immunohistochemistry
TCGA: The Cancer Genome Atlas
GEO: Gene Expression Omnibus
CCLE: Cancer Cell Line Encyclopedia
miRNAs: microRNAs
