# Supplementary material for: SNHG17 promotes colorectal tumorigenesis and metastasis via regulating Trim23-PES1 axis and miR-339-5p-FOSL2-SNHG17 positive feedback loop
Source: J Exp Clin Cancer Res. 2021 Nov 15;40:360. doi: 10.1186/s13046-021-02162-8 (PMC8591805; doi:10.1186/s13046-021-02162-8)
Supplement: Supplementary file 1 — Additional file 1. [file 13046_2021_2162_MOESM1_ESM.doc]

**Supplementary Tables**

**Supplementary Table S1. C**linicopathologic features of CRC patients

| **Characteristics** | **Colorectal cancer^a^**  **(n=142)** | **Colorectal cancer^b^**  **(n=107)** |
| --- | --- | --- |
| Ages (years) |  |  |
| < 65 | 87 | 75 |
| ≥ 65 | 55 | 32 |
| Gender |  |  |
| Male | 76 | 62 |
| Female | 66 | 46 |
| Tumor size (cm) |  |  |
| < 5 | 74 | 65 |
| ≥ 5 | 68 | 42 |
| Location |  |  |
| Colon | 70 | 58 |
| Rectum | 72 | 50 |
| Differentiation |  |  |
| Well and moderately | 124 | 89 |
| Poorly | 18 | 18 |
| Tumor stage |  |  |
| I+ II | 92 | 48 |
| III+IV | 50 | 59 |

^a^: SNHG17 expression data were available in 51 NCTs and 91 CRC tissues of cohort 1; PES1 expression data were available in 142 cases of cohort 1; FOSL2 expression data were available in 138 cases of cohort 1. ^b^: SNHG17 expression data were also available in 107 cases of cohort 2.

Supplementary Table S2. Primer sequences

| **Primers for real time PCR** | | |
| --- | --- | --- |
| **Primers** | **Sequences-F** | **Sequences-R** |
| SNHG17 | TGGGAGTGTCACATGACTGC | GCAGCTCAGCCTCTTCTTGA |
| β-actin | AGTGTGACGTGGACATCCGCAAAG | ATCCACATCTGCTGGAAGGTGGAC |
| U6 | CTCGCTTCGGCAGCACA | AACGCTTCACGAATTTGCGT |
| PES1 | GGGCATTTATCCCCATGAACC | CACCTTGTATTCACGGAACTTGT |
| FOSL2 | CAGAAATTCCGGGTAGATATGCC | GGTATGGGTTGGACATGGAGG |
| **Primers for PCR** | | |
| SNHG17-promoter-1 | TGGACCCTTGCAGAAAGAAGTC | TCAACGCCAGACTCTGGAGG |
| SNHG17-promoter-2 | GAGGCTGGAGTTTAGCAAAGC | AAGGCATCTGTCTTTGATTCAC |
| SNHG17-promoter-3 | AAGAGGACACCAGGGCTGTGT | CCACTTCCGCCATGATTGTAA |
| **Sequences for gene knockdown** | | |
| si-SNHG17-1 | CGTGTCTTCAAGAAGAGGCTGAGCT | AGCTCAGCCTCTTCTTGAAGACACG |
| si-SNHG17-2 | GATTGTCAGCTGACCTCTGTCCTGT | ACAGGACAGAGGTCAGCTGACAATC |
| si-SNHG17-3 | AAACGAGCGTAGCTTCCTTGT | ACAAGGAAGCTACGCTCGTTT |
| si-PES1-1 | CACATCATCAAGGAACGGTAT | ATACCGTTCCTTGATGATGTG |
| si-PES1-2 | GCATCACCCATCAGATTGT | ACAATCTGATGGGTGATGC |
| si-PES1-3 | CCAGAAGATCATGTTTGGCAA | TTGCCAAACATGATCTTCTGG |
| si-FOSL2-1 | GGATTATCCCGGGAACTTT | AAAGTTCCCGGGATAATCC |
| si-FOSL2-2 | GGAGTTCATGTTGGTGGCT | AGCCACCAACATGAACTCC |
| si-FOSL2-3 | CCACAGTGATCACCTCCAT | ATGGAGGTGATCACTGTGG |
| si-NC | TTCTCCGAACGTGTCACGT | ACGTGACACGTTCGGAGAA |
| **Primers for RNA pull-down** | | |
| SNHG17-Sense-F | TAATACGACTCACTATAGGGAGAGTATTTCCGCCGGCGCGA | |
| SNHG17-Sense-R | TGGAGCAATTCTGTAAGGTTTATTG | |
| SNHG17-Anti-F | GTATTTCCGCCGGCGCGA | |
| SNHG17-Anti-R | TAATACGACTCACTATAGGGAGATGGAGCAATTCTGTAAGGTTTATTG | |
| SNHG17-(1-750nt)-R | GTGCCCACAAACACCACCAAG | |
| SNHG17-(1-500nt)-R | CATGGGGCGGCAGTCATG | |
| SNHG17-(750-1037nt)-F | TAATACGACTCACTATAGGGAGAGAGAGGGGCAGAGAATGGAGA | |
| SNHG17-(5000-1037nt)-F | TAATACGACTCACTATAGGGAGATGTGTGAGAGGCGTCCTCTGG | |
| **Primers for cloning** | | |
| Sh-SNHG17-F | CCGGGATTGTCAGCTGACCTCTGTCCTGTCTCGAGACAGGACAGAGGTCAGCTGACAATCTTTTTTG | |
| Sh-SNHG17-R | AATTCAAAAAAGATTGTCAGCTGACCTCTGTCCTGTCTCGAGACAGGACAGAGGTCAGCTGACAATC | |
| SNHG17-F | GTGAACCGTCAGATCGAATTCGTATTTCCGCCGGCGCGA | |
| SNHG17-R | TAATCCAGAGGTTGAGGATCCTGGAGCAATTCTGTAAGGTTTATTG | |
| SNHG17-promoter-F-XhoI | GCGTGCTAGCCCGGGCTCGAGCGATTACAGGCGTGAGCCA | |
| SNHG17-promoter-R-HindIII | CAGTACCGGAATGCCAAGCTTGTCTGCAGATTTCGAGAGATGGG | |
| PES1-FL-F-BamHI | GATTACGCTTCTAGGGGATCCATGGGAGGCCTTGAGAAGAAG | |
| PES1-FL-R-EcoRI | AGAATCGATGATATCGAATTCTCACTCCGGCCTTGCCTT | |
| FOSL2-F-BamHI | GATTACGCTTCTAGGGGATCCATGTACCAGGATTATCCCGGG | |
| FOSL2-R-EcoRI | AGAATCGATGATATCGAATTCTTACAGAGCCAGCAGAGTGGG | |

**Supplementary figure legends**


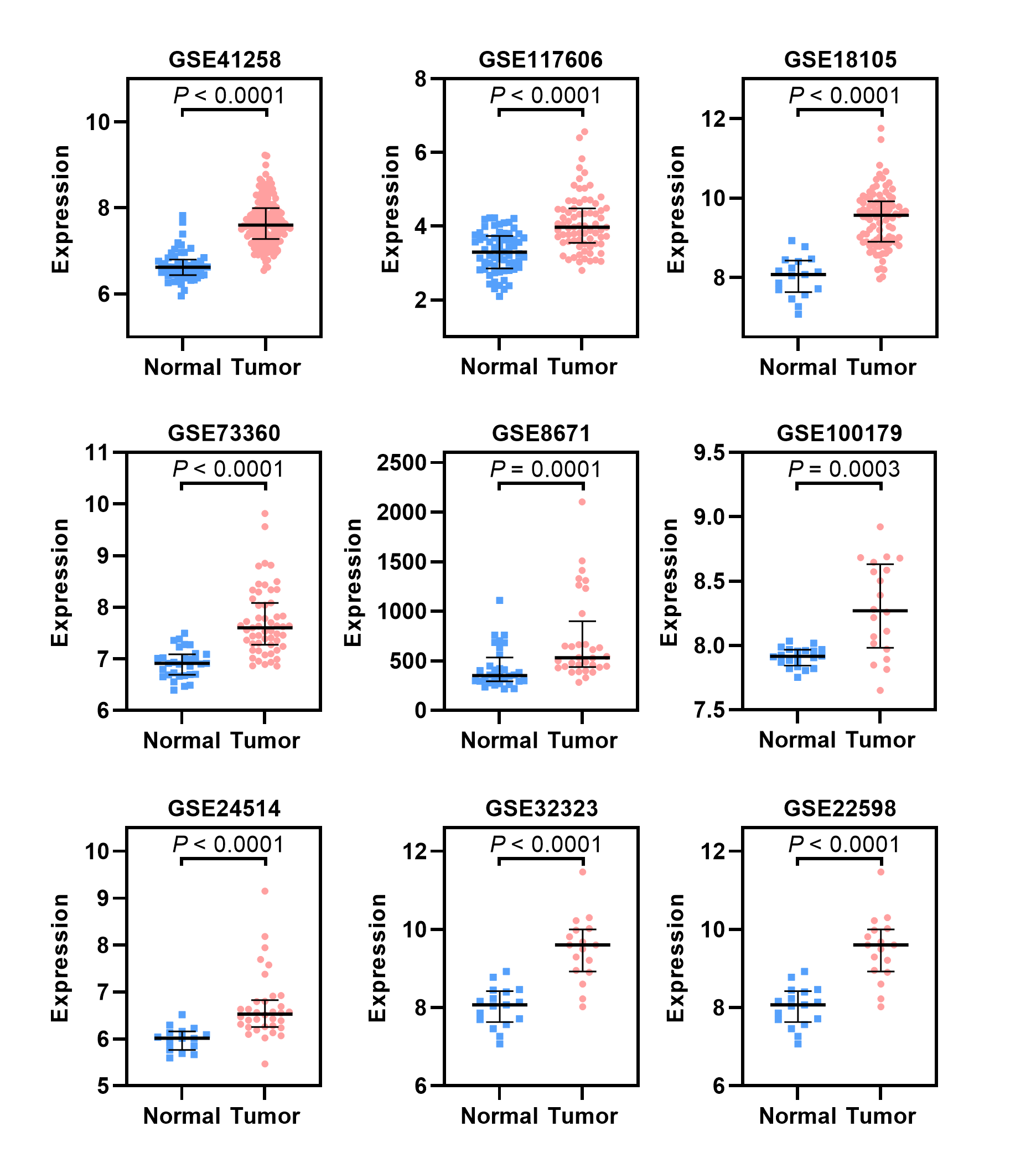


**Figure S1. Expression of SNHG17 in several CRC GEO databases.**

**
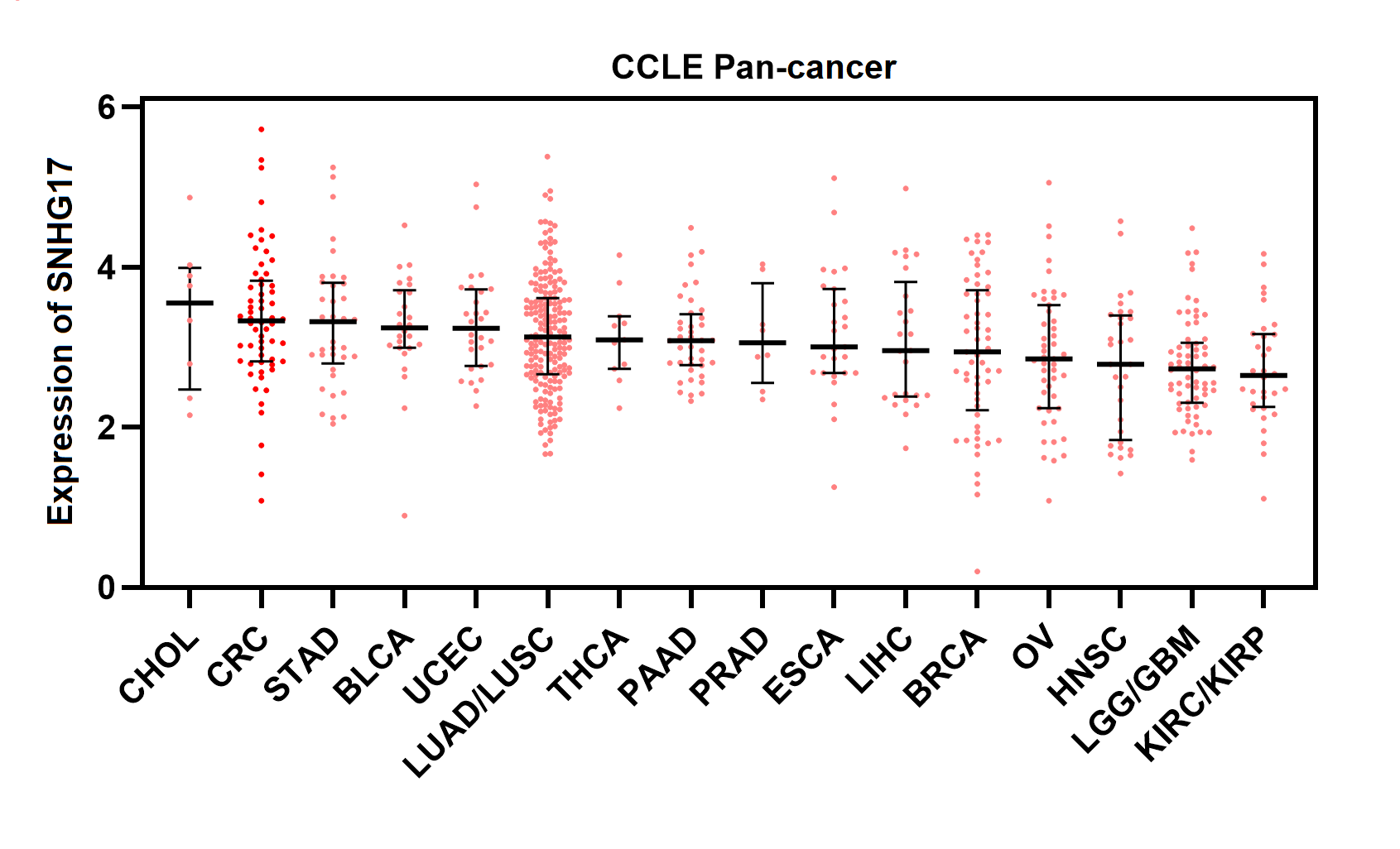
**

**Figure S2. Expression of SNHG17 in cell lines of CCLE database.**

**
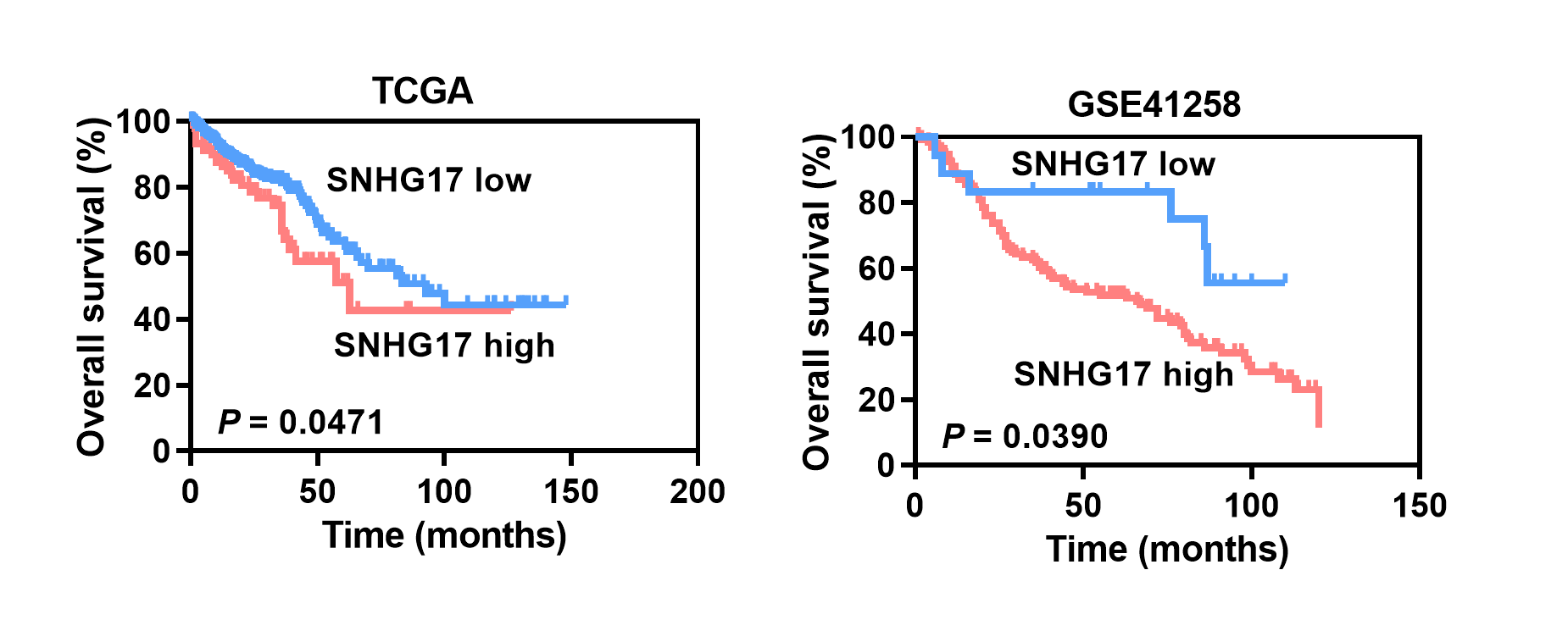
**

**Figure S3. Overall survival according to SNHG17 levels were analyzed using the Kaplan-Meier method in CRC datasets from TCGA and** **GSE41258.**

**
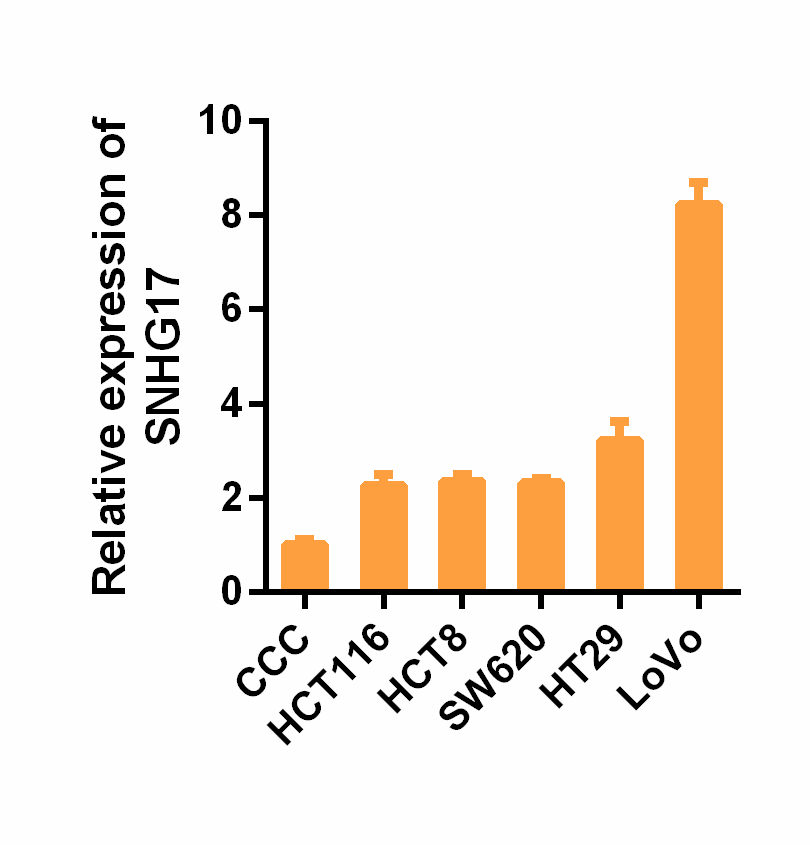
**

**Figure S4. Relative expression of SNHG17 in CRC cell lines was measured by RT-qPCR.**

**
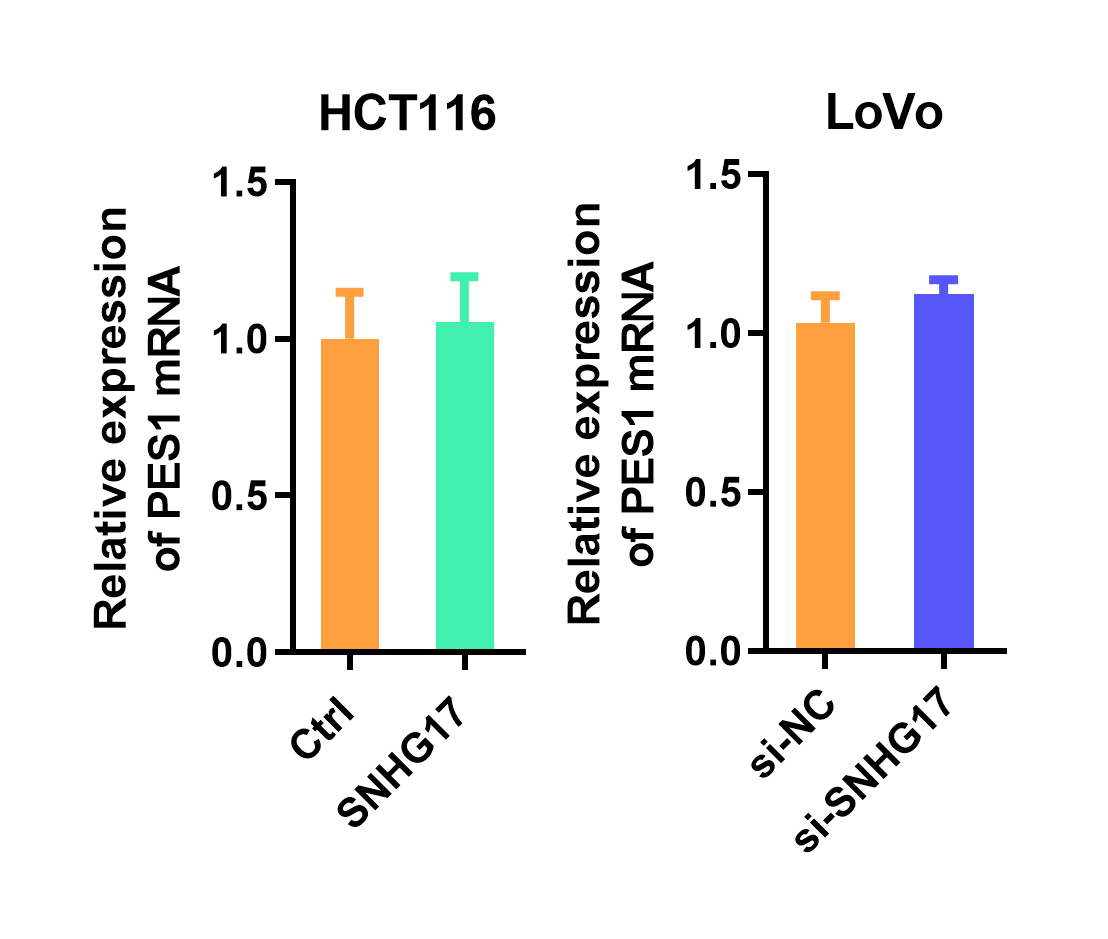
**

**Figure S5. The mRNA levels of PES1 were quantified by RT-qPCR in CRC cells with SNHG17 overexpression or knockdown.**

**
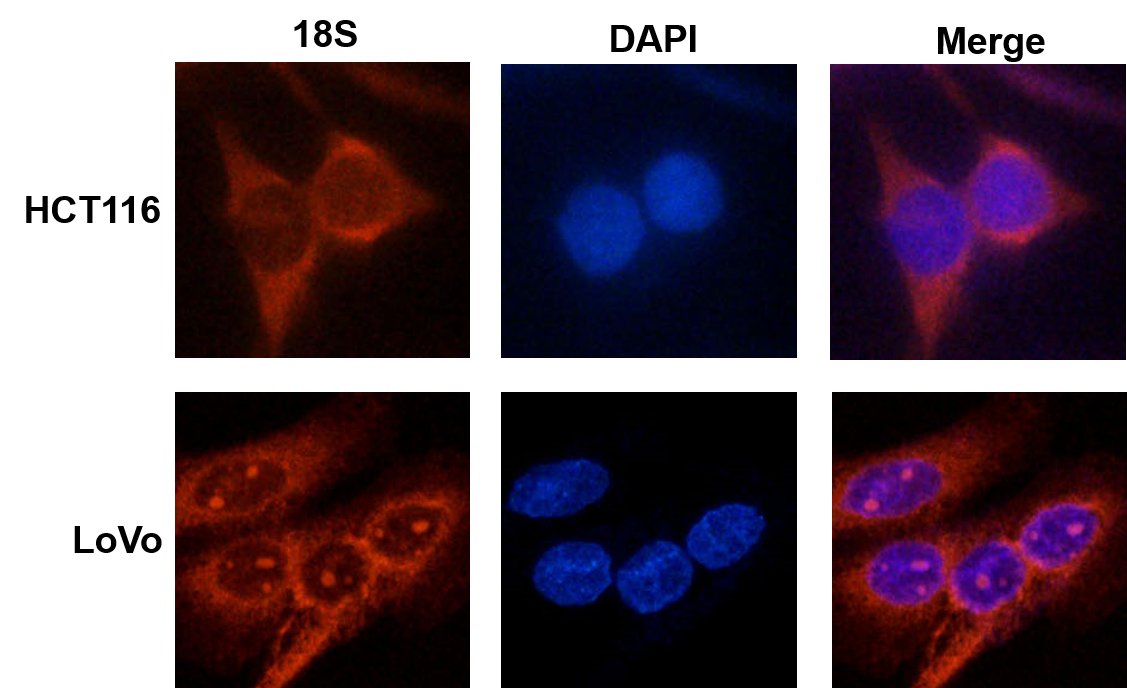
**

**Figure S6. 18S rRNA were used as positive controls for the cytoplasmic fractions of the FISH assay. DAPI-stained nuclei are blue.**

**
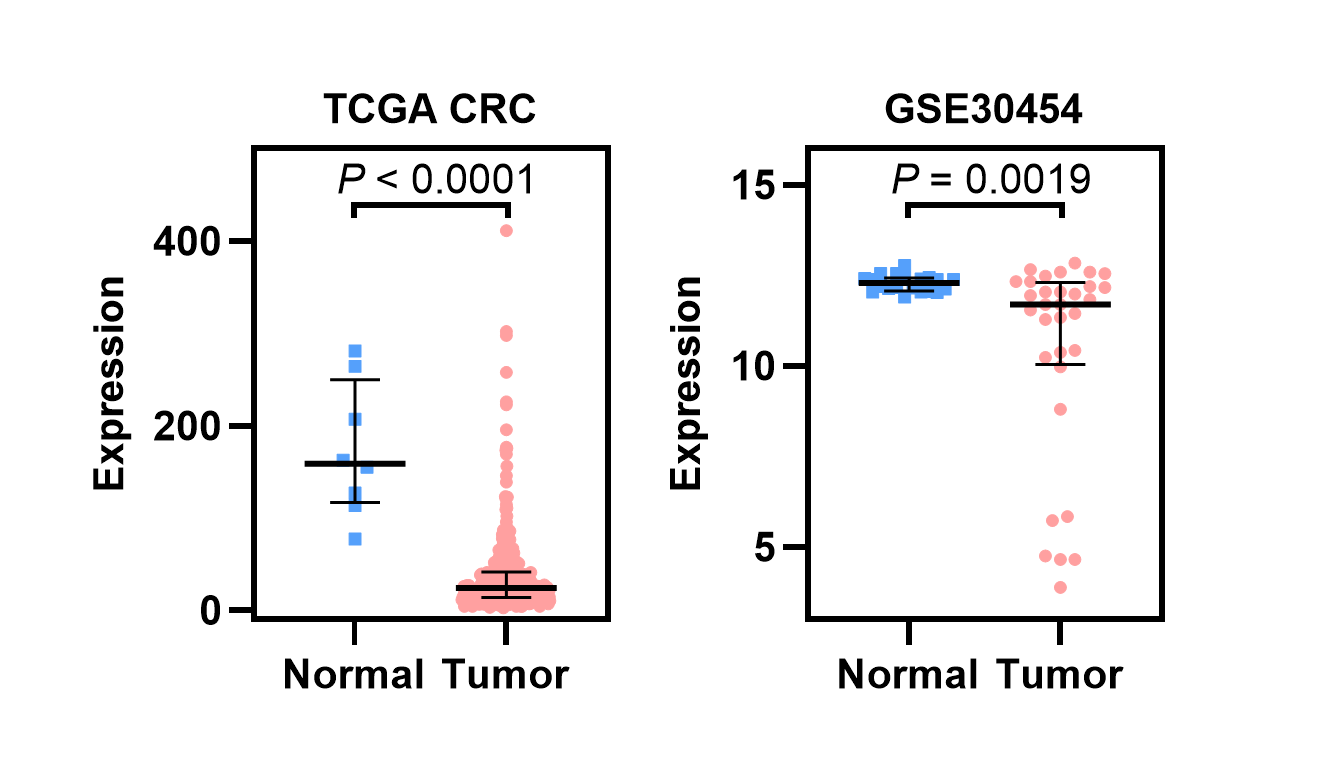
**

**Figure S7. Expression of miR-339-5p in CRC tissues of TCGA database and GSE30454 database.**


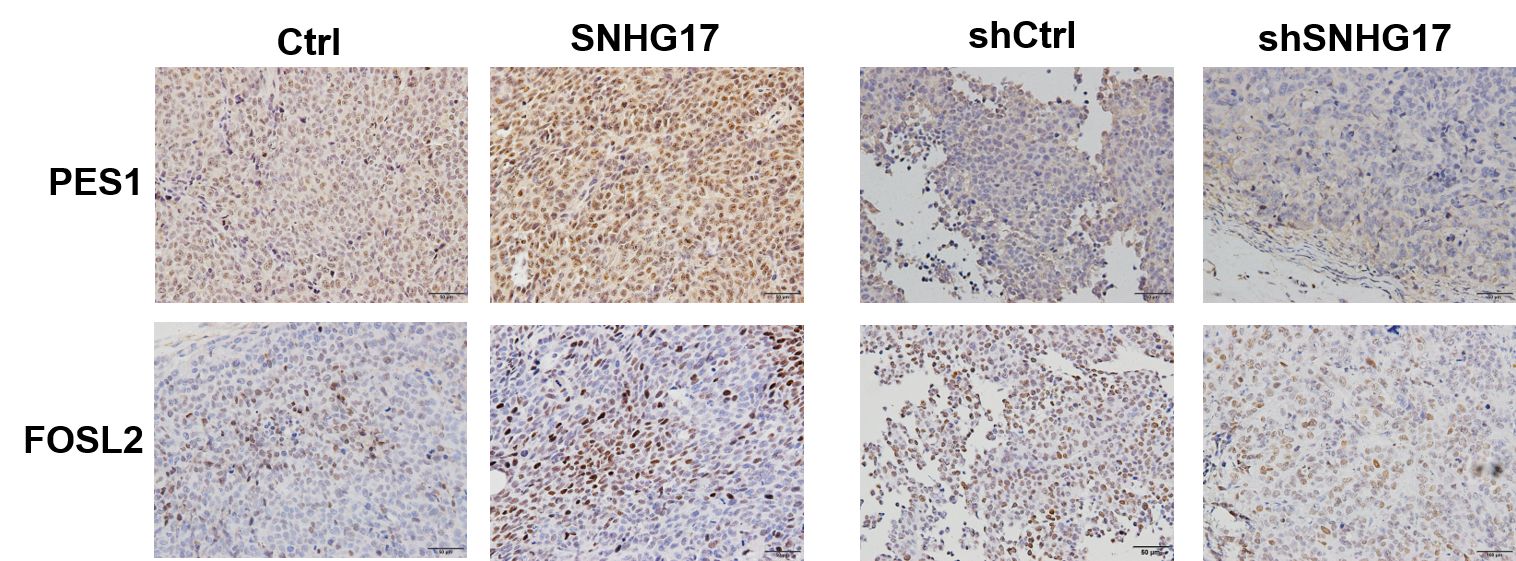


**Figure S8. The expression of PES1 and FOSL2 in SNHG17-overexpressed and -silenced CRC xenograft in nude mice were analyzed by IHC.**
